# Production and Characterization of an Extracellular Acid Protease from Thermophilic *Brevibacillus* sp. OA30 Isolated from an Algerian Hot Spring

**DOI:** 10.3390/microorganisms6020031

**Published:** 2018-04-12

**Authors:** Mohamed Amine Gomri, Agustín Rico-Díaz, Juan-José Escuder-Rodríguez, Tedj El Moulouk Khaldi, María-Isabel González-Siso, Karima Kharroub

**Affiliations:** 1 Equipe Métabolites des Extrêmophiles, Laboratoire de Recherche Biotechnologie et Qualité des Aliments (BIOQUAL), Institut de la Nutrition, de l’Alimentation et des Technologies Agro Alimentaires (INATAA), Université Frères Mentouri Constantine 1 (UFMC1), Route de Ain El Bey, 25000 Constantine, Algérie; gomrima@umc.edu.dz (M.A.G.); kkharroub@gmail.com (K.K.); 2 Grupo EXPRELA, Centro de Investigacións Científicas Avanzadas (CICA), Facultade de Ciencias, Universidade da Coruña, 15071 A Coruña, Spain; agustin.rico.diaz@udc.es (A.R.-D.); j.escuder@udc.es (J.-J.E.-R.); 3 Laboratoire Alimentation, Nutrition et Santé (ALNUTS), Institut de la Nutrition, de l’Alimentation et des Technologies Agro Alimentaires (INATAA), Université Frères Mentouri Constantine 1 (UFMC1), Route de Ain El Bey, 25000 Constantine, Algérie; moulouk.khaldi@umc.edu.dz

**Keywords:** *Brevibacillus* sp. OA30, thermophilic, hot spring, Algeria, protease, characterization

## Abstract

Proteases have numerous biotechnological applications and the bioprospection for newly-thermostable proteases from the great biodiversity of thermophilic microorganisms inhabiting hot environments, such as geothermal sources, aims to discover more effective enzymes for processes at higher temperatures. We report in this paper the production and the characterization of a purified acid protease from strain OA30, a moderate thermophilic bacterium isolated from an Algerian hot spring. Phenotypic and genotypic study of strain OA30 was followed by the production of the extracellular protease in a physiologically-optimized medium. Strain OA30 showed multiple extracellular proteolytic enzymes and protease 32-F38 was purified by chromatographic methods and its biochemical characteristics were studied. Strain OA30 was affiliated with *Brevibacillus thermoruber* species. Protease 32-F38 had an estimated molecular weight of 64.6 kDa and was optimally active at 50 °C. It showed a great thermostability after 240 min and its optimum pH was 6.0. Protease 32-F38 was highly stable in the presence of different detergents and solvents and was inhibited by metalloprotease inhibitors. The results of this work suggest that protease 32-F38 might have interesting biotechnological applications.

## 1. Introduction

Proteases catalyze the hydrolysis of proteinaceous material, and represent the largest worldwide enzyme sales [1]. Due to their characteristic active sites, in combination with their mode of catalytic action, proteases were assigned to groups of aspartic, cysteine, glutamic acid, serine, threonine, or metalloproteases. Moreover, they can be further subdivided based on their pH preferences into acidic, alkaline or neutral proteases [2]. Numerous commercial proteases, especially isolated from microorganisms, are used in various industrial and analytical processes, such as protein analysis, feed and food biotechnology, pharmaceutical and cosmetic preparations, and cleaning processes [3,4,5]. For example, they have major applications in detergent formulations, cheese-making, baking, meat tenderization, and leather industries [6,7,8].

Extracellular proteases produced by microorganisms are of great value for industry since they reduce production costs [9]. Thermophilic microorganisms are an important source of biodiversity and thermostable molecules of biotechnological importance and their unique properties at high temperatures justify the search for new proteases, as well as other enzymes of great value [10,11]. Thermostable proteases offer compatibility with processes that function more optimally at higher temperatures (e.g., through reduced viscosity), can have high catalytic efficiencies, and offer resistance from mesophilic microbial contamination [12]. Their robustness, in addition to their broad substrate specificity, makes thermostable proteases promising candidates for various industrial areas [13].

*Brevibacillus* belongs to the family Paenibacillaceae, a member of the Firmicutes phylum [14]. Among the 14 validated species of this genus, thermophilic *Brevibacillus thermoruber* and *Brevibacillus levickii* were isolated from different geothermal soils and hot springs [15,16]. These organisms have been reported to produce several molecules of biotechnological relevance, such as proteases, chitinases, exopolysaccharides, and bacteriocins, and to have the ability to be used as biocontrol agents and probiotics [17,18,19,20].

The aim of this study was to produce and characterize an extracellular protease from the thermophilic *Brevibacillus* sp. strain OA30 isolated from an Algerian hot spring.

## 2. Materials and Methods 

### 2.1. Isolation of Strain OA30

A water sample was collected from an Algerian hot spring located at Ouled Ali (36°34′ N; 7°23′ E) (54 °C; pH 7.0 ± 0.05). A total of 0.1 mL of the diluted sample was poured on Plate Counting Agar medium, (pH 7.2 ± 0.2) and incubated for 72 h at 55 °C. Strain OA30 was purified and replated on *Thermus* agar medium (% *w*/*v*: 3 agar; 0.8 peptone; 0.4 yeast extract; 0.2 NaCl; pH 7.2 ± 0.2) [21]. The chemicals used for this study were principally purchased from Sigma Chemical Co. (St. Louis, MO, USA), Merck and Co., Inc. (Kenilworth, NJ, USA), and Fluka Biochemika (Buschs, Switzerland). All media were sterilized at 120 °C for 20 min prior to inoculation.

### 2.2. Screening for Extracellular Proteolytic Activity

To reveal the extracellular proteolytic activity, strain OA30 was plated on casein agar plates (% *w*/*v*: 2.5 agar; 1.0 casein; 0.2 peptone; 0.1 yeast extract; 0.2 NaCl; pH 7.2 ± 0.2) and incubated at 55 °C for 48 h. The appearance of clear zones around the colonies confirmed the presence of the enzymatic activity [22]. 

### 2.3. Phenotypic Characterization

The phenotypic characterization of the isolate was performed by different tests referring to Bergey’s Manual of Determinative Bacteriology and minimal standards for describing new taxa of aerobic, endospore-forming bacteria [23,24]. The colonies’ aspect was examined. Cell morphology was observed using a light microscope (1000×, Leica DM 1000 LED (Leica Microsystems, Wetzlar, Germany)) fitted with a digital camera (Leica EC3 camera) after Gram staining of the cells. The presence of endospores was investigated using the Schaeffer-Fulton technique [23,25].

Requirements for NaCl were determined on *Thermus* agar medium at 0, 1, 3, 3.5, 5, 7.5, and 10% (*w*/*v*) NaCl. Growth was tested on pH values between 5 and 10 and on a temperature range between 30 and 75 °C. Different biochemical and physiological tests were also carried out: catalase and oxidase activities; indole and urease production; ONPG, Methyl Red (MR) and Voges–Proskauer (VP) reactions; fermentation and use as a carbon source of d-glucose, d-fructose, d-galactose, d-maltose, d-saccharose, and d-lactose; and hydrolysis of gelatin, pectin, and starch [26,27,28,29]. 

### 2.4. Estimation of Growth Rates

Growth rates were estimated at different temperatures, pH, and NaCl concentrations. Only one parameter was changed each time and the two other parameter values were kept constant. Table 1 shows the different value combinations used. To prepare the preculture, approximately 20 mL of *Thermus* liquid medium were inoculated with strain OA30 and incubated overnight at 55 °C. The preculture was then transferred into a sterile 500 mL flask containing 100 mL of the same modified *Thermus* liquid medium to give an initial absorbance at 660 nm of at least 0.1. The culture was incubated in aerobic conditions using a Thermo Scientific MaxQ 4000 Benchtop Orbital Shaker (Thermo Scientific, Waltham, MA, USA) at 120 rpm for approximately 24 h. At different time intervals, the turbidity of the cultures was determined by measuring the increase in optical density at 660 nm with a Synergy H1 hybrid multi-mode microplate reader. At least 10 absorbance measurements were taken into account.

### 2.5. Genotypic Characterization

#### 2.5.1. DNA Extraction, 16S rRNA Gene Amplification, and Sequencing

Strain OA was grown aerobically on *Thermus* medium agar (pH 7.2) at 55 °C for 24 h. Genomic DNA was extracted using a modified protocol described previously [30]. The quantity and quality of the genomic DNA was measured using a NanoDrop spectrophotometer (Thermo Scientific). The 16S rRNA gene was amplified by polymerase chain reaction (PCR) with universal bacterial primers E9F (GAGTTTGATCCTGGCTCA) [31] and U1510R (GGTTACCTTGTTACGACTT) [32]. A typical PCR contained (final concentration): 1× DreamTaq buffer, 1% (*v*/*v*) BSA (Bovine Serum Albumin), 1.25 U DreamTaq polymerase (Thermo Scientific), 1 µM (each) of primer, 200 µM of each deoxynucleoside triphosphate, and 10 to 100 ng of template DNA in a 50 mL reaction volume. PCR conditions were as follows: 95 °C for 3 min; 30 cycles of 95 °C for 30 s, 52 °C for 30 s, 72 °C for 85 s; and a final incubation at 72 °C for 5 min. PCR products were electrophoresed and visualized on a 1% (*w*/*v*) agarose gel. Amplicons were then purified with the GeneJET PCR purification kit (Thermo Scientific). E9F and U1510R primers were used for capillary sequencing at the Central DNA Sequencing Facility, University of Stellenbosch (South Africa).

#### 2.5.2. Phylogenetic Analysis

Identities with described taxa were investigated using the nBLAST tool against the EzBioCloud database of cultured organisms [33]. Multiple sequence alignments were performed using ClustalW [34]. 16S rRNA gene-based phylogenetic tree was constructed based on neighbor-joining [35] and maximum composite likelihood models [36] with 1000 bootstrap replications [37] using the MEGA 7 program package [38]. The sequence of *Sulfobacillus acidophilus* DSM 10332^T^ was used as the outgroup.

### 2.6. Enzyme Production

For the production of extracellular proteases, two different media were used: casein medium (M1) (% *w*/*v*: 0.8 casein, 0.3 peptone, 0.2 yeast extract, 0.2 glucose, optimum concentration of NaCl, 0.01 CaCl_2_·2H_2_O, 0.02 MgSO_4_·7H_2_O, 0.1 KH_2_PO_4_, optimum pH) and skim milk medium (M2) (% *w*/*v*: 8 skim milk, 0.3 peptone, 0.2 yeast extract, 0.2 glucose, optimum concentration of NaCl, 0.01 CaCl_2_·2H_2_O, 0.02 MgSO_4_·7H_2_O, 0.672 KH_2_PO_4_, 3.863 NaHPO_4_, optimum pH). A total of 50 mL of strain OA30’s preculture were prepared as in Section 2.4, and were used to inoculate 250 mL of medium M1 or medium M2 contained in a sterile 2000 mL flask. The culture was incubated at optimum temperature in vigorous aeration conditions at 140 rpm for 64 h. At different time intervals, the absorbance and the proteolytic activity of the cultures were determined as described in Section 2.9. Culture supernatants were collected by centrifugation (22,000× *g* for 30 min at 4 °C) and used as the crude enzyme solution.

### 2.7. Purification of Protease

Proteins from culture supernatants were filtered through 0.45 μm, then 0.2 µm pore sizemembrane filters, and the filtrates were precipitated by adding ammonium sulfate at a final concentration of 80% (*w*/*v*) and the suspension was kept at 4 °C overnight under gentle stirring. The precipitated proteins were collected by centrifugation at 22,000× *g* for 20 min at 4 °C and then dissolved in 25 mL phosphate-buffered saline (PBS) buffer (50 mM). The enzyme solution was dialyzed overnight in a 14 kDa cut-off dialysis tubing cellulose membrane at 4 °C against 500 mL of the same buffer, which was replaced four times every 2 h. The resulting solution was filtered again through 0.2 µm pore size membrane filters. 

Anion-exchange chromatography was performed using a HiTrap Q HP 5 mL column (GE Healthcare, Little Chalfont, UK). The column was equilibrated with 50 mM PBS (buffer A). Bounded proteins were eluted by applying a linear NaCl gradient (0–1 M) in buffer A and fractions were collected at 1 mL.

Active fractions were subsequently concentrated using a 10 kDa cut-off Amicon Pro Purification System (Millipore, Burlington, MA, USA). Tubes were first washed with 50 mM PBS. 

A second purification of the fractions with enzymatic activity was done by gel filtration using a HiLoad 16/60 Superdex 200 prepgrade column (GE Healthcare) in 50 mM PBS buffer. Fractions were collected at 1 mL.

### 2.8. Molecular Weight Determination and Zymography

The SDS-PAGE method in a 10% polyacrylamide slab gel was carried out to analyze the molecular mass [39]. For zymogram analysis, protease was separated in a 10% SDS-polyacrylamide gel containing 0.5% (*w*/*v*) skim milk as a substrate. The samples were not heated prior to electrophoresis. Electrophoresis of both gels was run at 150 V for 120 min at room temperature. The zymography gel was washed with 2.5% (*v*/*v*) Triton X-100 for 1 h and incubated for 15 min in 50 mM Tris-HCl buffer, pH 7.5, and was then incubated at 50 °C overnight in a PBS solution (pH 7.5) containing 1% (*w*/*v*) casein. The gel was stained with Coomassie Brilliant Blue R-250 (0.2% *w*/*v*) for 1 h and then destained in distilled water/acetic acid (80:20). The protease band appeared as a clear zone surrounded by the blue color of the gel. NZY Color Protein Marker II (Lisbon, Portugal) was used as a molecular marker for both electrophoresis techniques and for the estimation of the molecular weight of protease.

### 2.9. Enzyme Assay

Protease activity was determined using azocasein as a substrate. The reaction was performed in 50 mM PBS solution at pH 7.5 with 50 µL of azocasein (30 mg/mL in water) and with 25 µL of the enzyme solution for a final volume of 750 µL. The reaction was incubated in the dark at 50 °C for 1 h and stopped by adding 125 µL of 20% (*w*/*v*) trichloroacetic acid. The blank assay was performed using 25 µL of culture medium or PBS buffer. After centrifugation at 15,000× *g* for 10 min, the absorbance of the supernatant was measured at 366 nm using a Synergy H1 Hybrid Multi-Mode Microplate Reader [40]. One unit of protease activity was defined as the amount required to produce enough acid-soluble material from azocasein to yield an absorbance of 0.01 at 366 nm, following 1 h of incubation.

Protein was quantified by the Bio-Rad protein assay kit (Hercules, CA, USA) [41] with BSA as the standard.

### 2.10. Effect of Temperature on Protease Activity and Stability

The optimum temperature was determined by measuring enzyme activity at 30–80 °C as described above. Enzyme stability was measured by incubating for 20, 30, 40, 50, 60, 90, 120, 180, and 240 min at optimum temperature in 50 mM PBS buffer pH 7.5.

### 2.11. Effect of pH on Protease Activity 

The effect of pH on the enzymatic assay was determined by measuring the enzymatic activity using substrate solutions with different pH (4–11; Na_2_HPO_4_-citric acid: 5.0–7.0; Tris-HCl: 8.0–9.0; and glycine-NaOH: 10.0–11.0) at optimum temperature.

### 2.12. Effect of Metal Ions on Protease Activity

The effect of various metal ions on enzyme activity was determined by incubating the enzyme with 2.5 mM metal ions (Mg^2+^, Li^2+^, Fe^3+^, Cu^2+^, Zn^2+^, Mn^2+^, and Ca^2+^). The protease activity without metal ions served as the control and was considered as 100% activity.

### 2.13. Effect of Solvents on Protease Activity

The effect of solvents ethanol, methanol, and acetone on protease activity was measured by incubating the enzyme with 1% (*v*/*v*) of the solvents. The protease activity without solvents served as the control, which was considered as 100% activity.

### 2.14. Effect of Detergents and Chemicals on Protease Activity

The effect of different concentrations of surfactants: SDS, Tween-20, Tween-80, Triton X-100, metal ion chelators ethylenediaminetetraacetic acid (EDTA), dithiothreitol (DTT), protease inhibitors phenylmethylsulfonyl fluoride (PMSF), pepstatin A, trypsin inhibitor and dimethyl sulfoxide (DMSO) were studied by incubating enzyme with 1% of chemical or 1 mM of EDTA, trypsin inhibitor, and pepstatin A. The protease activity without chemicals served as the control, which was considered as 100% activity.

### 2.15. Statistical Analysis

Three replicates of each sample were used for statistical analysis using STATISTICA 12 software [42]. Statistical analysis was conducted by Student’s t test. A probability level of *p* < 0.05 was considered statistically significant.

## 3. Results and Discussion

### 3.1. Isolation and Characterization of Protease-Producing Strain OA30 

Strain OA30 was isolated on a nutritive agar medium used for the isolation of aerobic heterotrophic bacteria, from water samples of an Algerian terrestrial hot spring with moderate temperature (54 °C), chloride–calcica water type [43], and neutral pH (7.0). Screening for extracellular protease activity on casein agar was positive. Large, clear zones appeared around the colonies indicating the production of an extracellular enzymatic activity against casein. 

#### 3.1.1. Phenotypic Characterization

**Figure 1:**
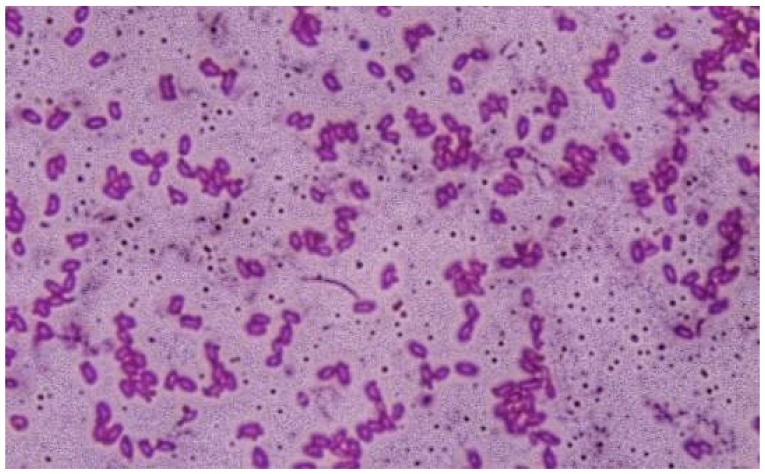
Cells of strain OA30 viewed by light microscopy (1000×) after Gram staining.

Strain OA30 produced smooth, flat, spreading colonies, with yellowish color and no particular pigmentation on *Thermus* medium. Cells were rod-shaped, Gram-positive (Figure 1), and motile, with the presence of terminally-born ellipsoidal spores. The strain was aerobic, catalase, and oxidase positive. It was able to use all the tested sugars as carbon sources, but was unable to ferment them and could not use citrate. ONPG, VP, and MR reactions were negative. The strain was unable to produce indole, but it could use urea and hydrolyze, in addition to casein, gelatin, and starch, but did not degrade pectin.

**Figure 2:**
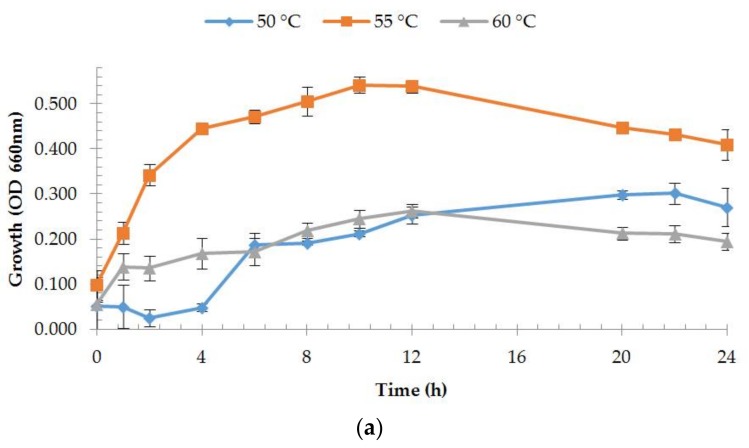

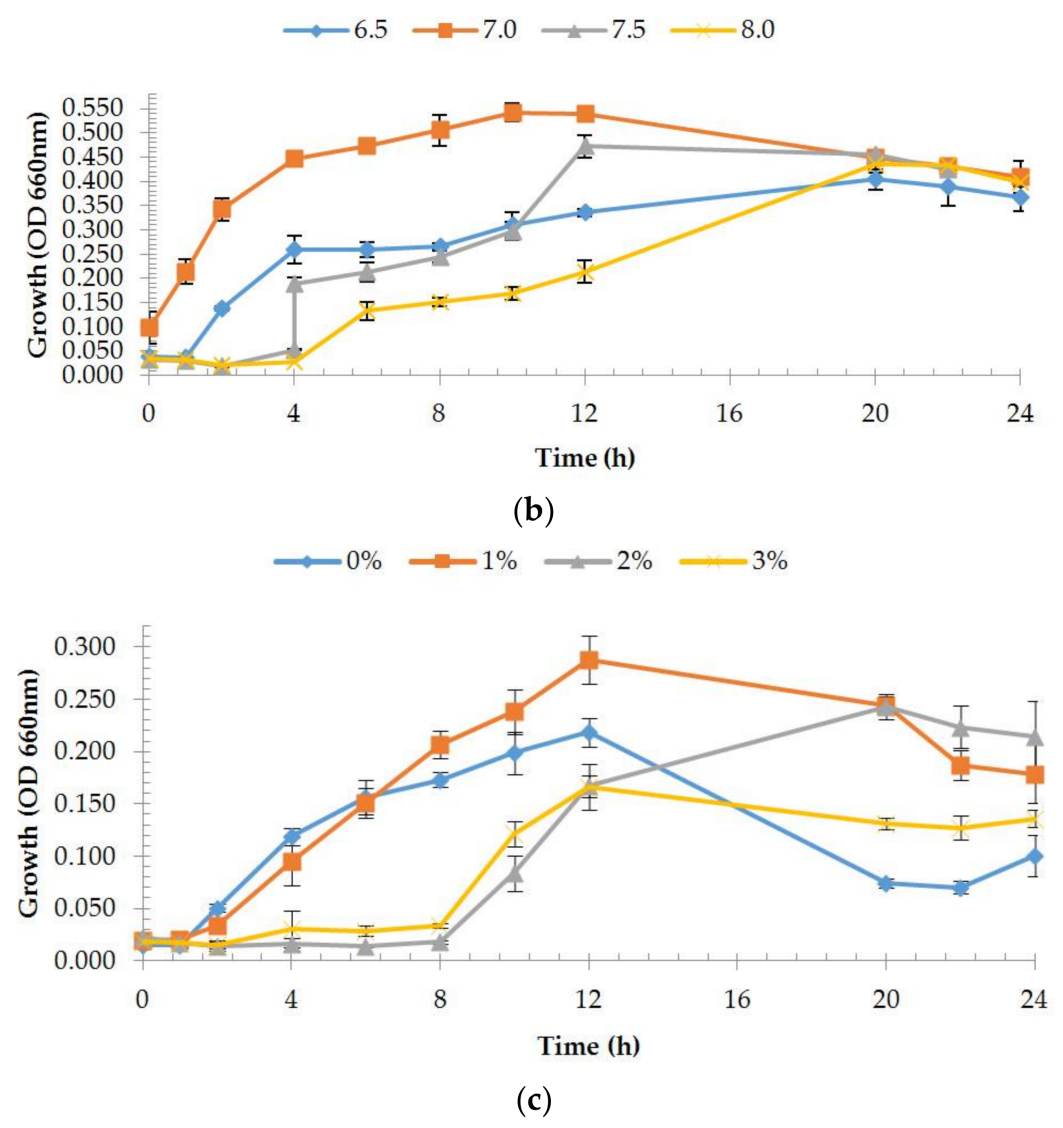
Growth of strain OA30 under different physiological conditions: (**a**) growth at different temperatures; (**b**) growth at different pH values; and (**c**) growth at different NaCl concentrations.

The growth of strain OA30 occurred at 30–70 °C, a pH range from 6.0 to 8.6, and was stopped by a 5% concentration of NaCl. Optimal growth on liquid medium was studied by measuring the absorbance of the cultures under variable physiological conditions and significantly occurred at 55 °C, pH 7.0, and at a concentration of NaCl of 1% (*w*/*v*) (Figure 2). These values were very close to those from the isolation site and were used as parameters to optimize the growth medium for protease production.

#### 3.1.2. Genotypic Characterization

**Figure 3:**
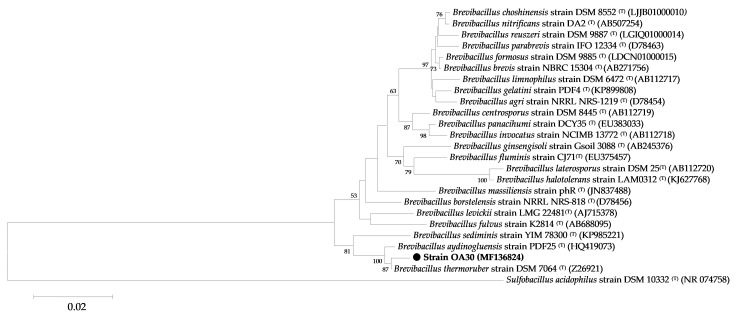
Phylogenetic tree based on 16S rRNA gene sequences showing the relationship between strain OA30 (1489 bp) and strains of the genus *Brevibacillus*. The strains and their corresponding Genbank accession numbers are shown following the organism name and indicated in parentheses. The phylogenetic tree was made using the neighbor-joining method with maximum composite likelihood model implemented in MEGA 7. The tree includes the 16S rRNA gene sequences of *Sulfobacillus acidophilus* DSM 10332^T^ as an outgroup. Bootstrap consensus trees were inferred from 1000 replicates, and only bootstrap values >50% are indicated. The scale bar represents 0.02 nucleotide changes per position. (●) indicates the isolate assessed in the current study, *Brevibacillus* sp. strain OA30.

The 16S rRNA gene sequences of strain OA30 have been deposited in the NCBI database under accession number MF136824. Based on the 16S rRNA gene sequence similarity searches by the nBLAST tool against the EzBioCloud database, strain OA30 showed 92 to 99% sequence similarity to members of the genus *Brevibacillus*. A 16S rRNA gene-based phylogenetic tree of *Brevibacillus* sp. strain OA30 was constructed (Figure 3). The *Brevibacillus* sp. strain OA3016S rRNA gene sequence exhibited high identity (99%) with type strain *Brevibacillus thermoruber*, strain DSM 7064^T^ (Z26921), the closest validly published *Brevibacillus* species.

### 3.2. Protease Purification

#### 3.2.1. Enzyme Production and Medium Composition Effect

**Figure 4:**
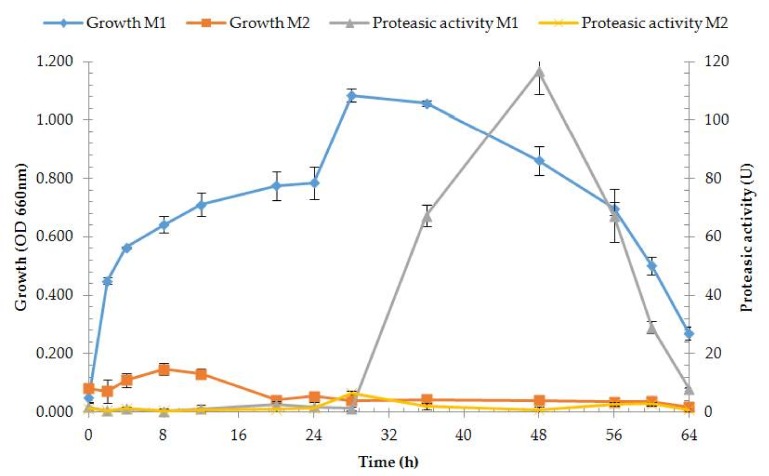
Protease activities and growth rates of strain OA30 on M1 and M2.

Extracellular protease production assay was performed on two different media, M1 (casein) and M2 (skim milk) (at 55 °C, pH 7.0, NaCl 1% *w*/*v*). Good enzyme activity and growth rates were obtained on M1, while growth on M2 was very low with no enzyme production, which appears to be unsuited for growth of strain OA30 (Figure 4). Extracellular protease activity appeared on M1 after 36 h of cultivation with the best, significantly higher value after 48 h. Purification was consequently performed on the crude enzyme solution collected from M1 (Table 2).

#### 3.2.2. Protease Precipitation, Anion Exchange, and Gel Filtration Chromatography 

**Figure 5:**
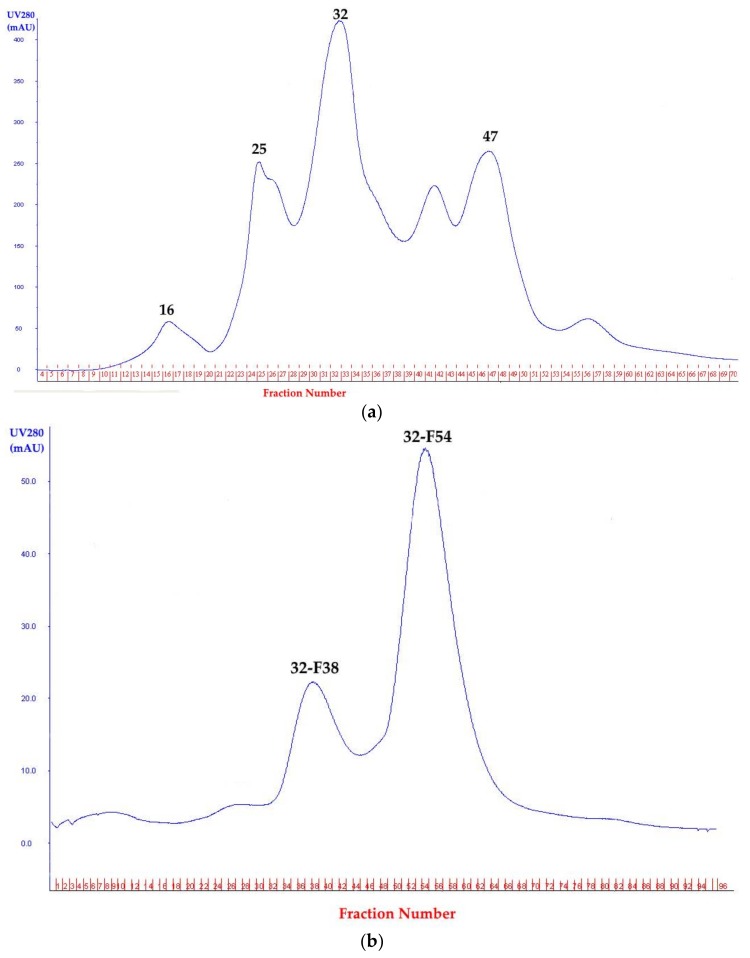
Purification of extracellular protease from the culture supernatant of strain OA30. (**a**) Anion-exchange chromatography on a HiTrap Q HP column of the dissolved precipitate from the culture supernatant. Protease activity was detected in fractions 16, 25, 32, and 47. Fraction 32 was selected for a second purification step; and (**b**) gel filtration chromatography on a HiLoad 16/60 Superdex 200 prepgrade column of fraction 32 containing the highest protease activity from anion-exchange chromatography. Protease activity was detected in peaks 32-F38 and 32-F54 and protease 32-F38 was selected for further investigation.

Four fractions with protease activity were obtained after first purification with a HiTrap Q HP column. Fraction 32 had the highest enzyme activity (11.79 U/mg) (Figure 5a) and was selected for a second round of purification with the HiLoad 16/60 Superdex 200 prepgrade column, which gave two fractions with protease activity. Fraction 32-F38 had, significantly, the best activity (Figure 5b). This protease, named protease 32-F38, was examined for further characterization (Table 2). The presence of multiple extracellular proteases was reported for *Brevibacillus* strains isolated from similar environments. Thermophilic *Brevibacillus* species are well-known hydrolase producers [24,44].

### 3.3. Molecular Weight Determination and Zymoraphy

**Figure 6:**
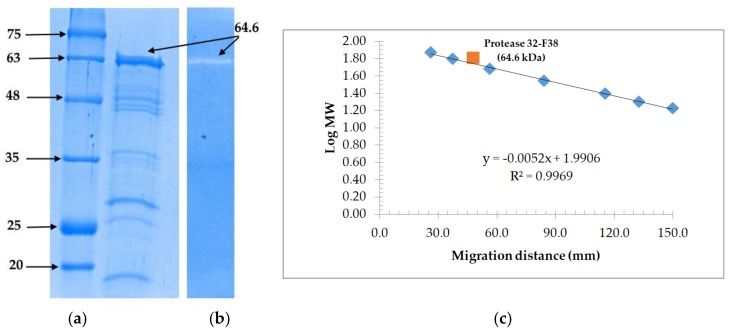
Electrophoresis analysis and purification of protease 32-F38 from strain OA30. (**a**) SDS-PAGE of the purified protease. Lane 1, protein markers (kDa). Lane 2, partially-purified protease 32-F38 obtained after gel filtration; (**b**) Zymogram activity staining of the purified protease; (**c**) Estimation of the molecular mass of protease 32-F38.

Electrophoretic profiles of fraction 32-F38 were studied. Zymography revealed protease activity for the band corresponding to an estimated molecular weight of 64.4 kDa on the corresponding SDS-PAGE gel led in the same migration conditions for fraction 32-F38 after gel filtration chromatography (Figure 6). Monomeric proteases with molecular weights between 60 and 66 kDa from *Brevibacillus* sp. have been reported in literature [45,46,47].

### 3.4. Biochemical Characterization

#### 3.4.1. Effect of Temperature and Thermostability of the Enzyme

**Figure 7:**
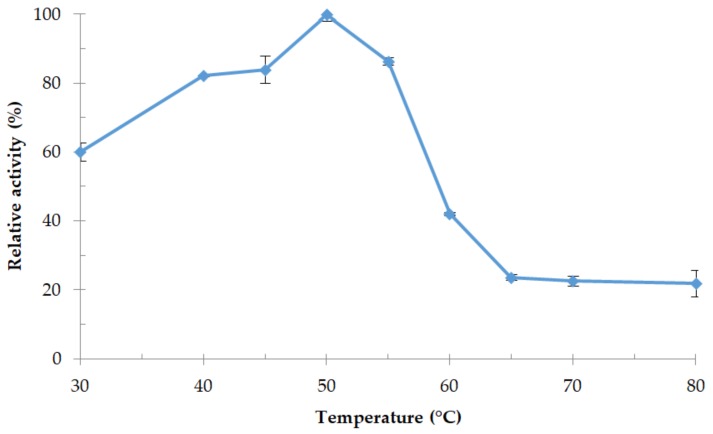
Optimum temperature of protease 32-F38 activity. Relative activity is expressed as a percentage of the maximum.

**Figure 8:**
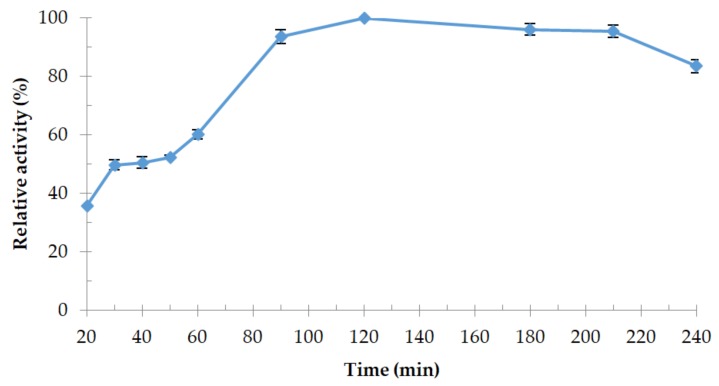
Thermostability of protease 32-F38 at optimum temperature (50 °C). Relative activity is expressed as a percentage of the maximum (activity after 120 min).

Relative activity of protease 32-F38 at different temperatures is shown in Figure 7. Protease activity was significantly higher at 50 °C, so this temperature was considered as the optimum temperature for the enzyme and was similar to the optimum growth temperature of strain OA30. Protease 32-F38 remained at least 80% active in the range between 40 °C and 55 °C. Thermostability tests at 50 °C revealed that the activity was the highest after 120 min of heating and relative activity lost only 16% of its value after 240 min (Figure 8). It is not uncommon that relative enzymatic activity increases after short-term heating in the case of thermoactive enzymes, as it happens with protease 32-F38 (Figure 8), and examples of other proteases can be cited [48,49].

#### 3.4.2. Effect of pH 

**Figure 9:**
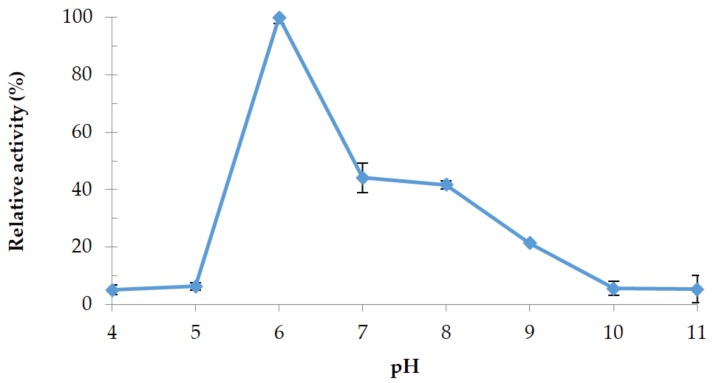
Effect of pH on protease 32-F38 activity. Relative activity is expressed as a percentage of the maximum.

The effect of pH buffers is illustrated in Figure 9. Protease 32-F38 was found to be an acid protease; optimum pH was 6.0 in Na_2_HPO_4_-citric acid buffer with a relative activity 55% and 58% higher than at pH 7.0 and pH 8.0 in Tris-HCl buffer, respectively. Weak activities were still observed at pH 5.0 and 11.0. The majority of extracellular proteases reported in literature from *Brevibacillus* members were alkaline proteases with optimal pH around 8.0 [46,47,50,51], and acid proteases were rarely reported [45]. 

#### 3.4.3. Effect of Various Chemicals on Protease 32-F38 Activity

Relative activities of protease 32-F38 in the presence of different chemical agents are shown in Table 3. Ions of Mg^2+^ and Mn^+2^ showed a significant enhancing effect on the enzymatic activity with the best effect with magnesium ions, which was reported for other proteases from *Brevibacillus* spp. [50,51], and might have a protective effect against thermal denaturation of the enzyme [52]. Li^2+^ had no significant effect on protease 32-F38 while a slight inhibition was noted with calcium ions. Protease activity was completely inhibited by Zn^2+^, Cu^2+^, and Fe^3+^. The same inhibitory effect of heavy metal ions was observed on a thermostable protease of several *Brevibacillus* species and is probably related to a reaction with the protein thiol groups (converting them to mercaptides), as well as with histidine and tryptophan residues. Metal ions are potent inhibitors of protein folding [46,47,51]. Protease 32-F38 activity was not affected by a 1% concentration of methanol, ethanol, or acetone. The addition of 1% SDS caused a four-fold increase in the enzyme activity, and SDS is known to denature protein substrates, such as casein, resulting in increased digestion rates. The remarkable resistance of protease 32-F38 toward SDS denaturation was also observed for other thermoactive proteases, like proteinase K and thermopsin, and might be related to a modification of the protein structure due to a high number of tyrosine residues [53,54,55]. Enzyme activity was significantly enhanced by 1% of Tween-80 and Triton X-100 while the presence of Tween-20 was strongly inhibiting the enzyme activity. The presence of metalloprotease inhibitors EDTA and DTT inhibited the protease activity completely and 1% of DMSO reduced it by 12%. On the other hand, the inhibitor of aspartyl proteases, pepstain A, and the inhibitors of serine proteases, trypsin inhibitor, and PMSF, increased the enzyme activity significantly. These results indicate that protease 32-F38 is most probably a metalloprotease [56,57].

## 4. Conclusions

Geothermal sites are an important source of valuable molecules and only a few studies were interested in the biotechnological potential of thermophilic microorganisms isolated from Algerian hot springs. *Brevibacillus* sp. strain OA30 studied in this work showed multiple extracellular protease activities that should be investigated more closely with biochemical and genomic methods. 

The characterization of the purified protease 32-F38 revealed interesting abilities of the enzyme in acid conditions and in the presence of solvents and detergents with a high stability at 50 °C. Protease 32-F38 seems to be related to other metalloproteases, such as thermolysin (EC 3.4.24.27) and bacillolysin (EC 3.4.24.28) produced by several strains of the aerobic thermophilic genera *Bacillus*, *Geobacillus* and *Brevibacillus*. Thermolysin-like proteases have potential applications in the degradation of gelatin, keratin, and other raw materials, like wheat bran and fish scales in biotechnological applications [19]. They can also act as peptide and ester synthetases and can be used for peptide synthesis and the production of a precursor of the artificial sweetener aspartame [58], and have been used for the hydrolysis of plant cell walls in order to assist in aqueous extraction processes [59]. Proteases with similar characteristics and molecular weight also showed anti-biofilm activity against pathogenic bacteria such as *Listeria monocytogenes*, *Escherichia coli*, and *Salmonella typhi* [49] and are potential candidates for the production of bioactive peptides from casein [60]. Our initial promising results contribute towards the application of protease 32-F38 in industrial and analytical processes.

## Figures and Tables

**Table 1:** Temperature, pH and NaCl concentration values used to estimate growth rates.

	Assay	1	2	3	4	5	6	7	8	9	10
Parameter											
T (°C)		50	55								60
pH *		7.0	7.0	6.5	7.0	7.5	8.0	7.0	7.0	7.0	7.0
[NaCl] (% *w*/*v*)		0						1	2	3	0

* Phosphate buffer (0.2 M) was used to adjust the pH values.

**Table 2:** Purification summary of protease 32-F38 from *Brevibacillus* sp. strain OA30.

Purification Step	Total Protein (mg)	Total Activity (U)	Specific Activity (U/mg)	Yield (%)	Purification (Fold)
Cell-free supernatant	131.78	147.2	1.12	100.00	1.00
80% ammonium sulfate	32.09	130.1	4.05	88.38	3.63
Dialysis	11.90	128.1	10.76	87.02	9.64
AE chromatography	2.54	30.0	11.79	20.38	10.56
Gel filtration	1.68	27.4	16.31	18.61	14.60

**Table 3:** Effects of various metal ions, solvents, detergents, and other chemicals on protease from fraction 32-F38 stability.

Reagent	Concentration	Relative Activity ^1^ (%)
Mg^2+^	2.5 mM	143.24 ± 2.13
Li^2+^	2.5 mM	100.29 ± 3.25
Fe^3+^	2.5 mM	0.00 ± 0.00
Cu^2+^	2.5 mM	7.35 ± 0.00
Zn^2+^	2.5 mM	0.00 ± 0.00
Mn^2+^	2.5 mM	113.24 ± 1.85
Ca^2+^	2.5 mM	90.89 ± 3.45
Ethanol	1%	96.46 ± 2.06
Methanol	1%	106.19 ± 2.47
Acetone	1%	103.54 ± 3.06
SDS	1%	438.35 ± 3.56
Tween-20	1%	27.73 ± 2.95
Tween-80	1%	144.54 ± 4.25
Triton X-100	1%	144.25 ± 3.12
EDTA	1.0 mM	0.88 ± 0.26
DTT	1%	0.36 ± 0.02
PMSF	1%	193.59 ± 3.15
Pepstatin A	1.0 mM	111.39 ± 3.23
Trypsin inhibitor	1.0 mM	186.48 ± 2.23
DMSO	1%	87.90 ± 3.56

^1^ The activity is expressed as a percentage of the activity level in the absence of reagent.
